# Draft Genome Sequence of *Streptococcus agalactiae* PR06

**DOI:** 10.1128/genomeA.00351-13

**Published:** 2013-06-13

**Authors:** Irma Syakina MZ, L. K. Teh, M. Z. Salleh

**Affiliations:** Pharmacogenomics Centre (PROMISE), Faculty of Pharmacy, Universiti Teknologi MARA Malaysia, Puncak Alam Campus, Selangor Darul Ehsan, Malaysia

## Abstract

*Streptococcus agalactiae* (group B streptococcus [GBS]) is a Gram-positive bacterium that was first recognized as a causative agent of bovine mastitis. *S. agalactiae* has subsequently emerged as a significant cause of human diseases. Here, we report the draft genome sequence of *S. agalactiae* PR06, which was isolated from a septicemic patient in a local hospital in Malaysia.

## GENOME ANNOUNCEMENT

*Streptococcus agalactiae* is well-adapted to asymptomatic colonization in the gastrointestinal and genitourinary tracts of healthy individuals (1). However, under certain circumstances, it may turn into a life-threatening pathogen causing septicemia, meningitis, and pneumonia in neonates (2). It is also a serious cause of mortality and morbidity in nonpregnant adults, particurly in elderly persons and those with underlying diseases (3). Here, we report the draft genome sequence of the *S. agalactiae* PR06 isolate from a local hospital in Malaysia.

*S. agalactiae* PR06 was grown in Todd-Hewitt broth under optimal growth conditions. Gram stain and biochemical tests were performed to determine the purity of the bacterium. The genomic DNA of *S. agalactiae* PR06 was isolated using the Wizard genomic DNA purification kit (Promega). The draft genome of local *S. agalactiae* PR06 was determined using the Genome Analyzer IIx platform (Illumina). This genome was sequenced to produce ultra-deep coverage of 1,000×, and the quality was checked using FastQC. The reads were trimmed using the CLC bio Genomic Workbench 5.1 and assembled by mapping against the genome of the closest reference strain, *S. agalactiae* A909. A total of 67 contigs were produced, and these contigs were ordered with respect to the best-aligned positions compared to the reference genome of *S. agalactiae* A909 using Optimal Syntenic Layout of Unfinished Assemblies (OSLay) (4). The genome was annotated using the Rapid Annotations using Subsystems Technology (RAST) server (5), and putative coding sequences (CDSs) and annotated genes were identified by comparing outputs from the Bacterial Annotation System (BASys) (6).

The draft genome of *S. agalactiae* contains a circular chromosome of 2,120,750 bp with a G+C content of 37% and 2,143 protein-coding sequences. The genome revealed >37 RNA genes and several virulence genes, including the *cyl* locus, which is responsible for pulmonary epithelial cell damage; the *cfb* gene, which codes for CAMP factor (7); the *scp* gene, which codes for C5a peptidase (8); and gene loci involved in capsule synthesis.

These observations and comparative genomic studies would be useful in providing better knowledge of the genetic and molecular elements that might reveal drug-resistance profiles and vulnerabilities. The genome offers targets for the development of diagnostic tests, as well as new antimicrobial drugs and vaccines.

### Nucleotide sequence accession number.

The genome sequence of *S. agalactiae* PR06 has been deposited at DDBJ/EMBL/GenBank under the accession no. AOSD00000000.
